# Supplementary material for: Development of a computer-aided design software for the quantitative evaluation of aesthetic damage
Source: PLoS One. 2019 Dec 18;14(12):e0226322. doi: 10.1371/journal.pone.0226322 (PMC6919621; doi:10.1371/journal.pone.0226322)
Supplement: S1 Code site — 1. (ZIP) [file pone.0226322.s001.zip › [EESC_jr][Projeto]Relatorio_07_21/vendors/select2/docs/_includes/examples/multiple-max.html]

# Limiting the number of selections

Select2 multi-value select boxes can set restrictions regarding the
maximum number of options selected. The select below is declared with
the `multiple` attribute with `maximumSelectionLength`
in the select2 options.

{% highlight js linenos %}
$(".js-example-basic-multiple-limit").select2({
maximumSelectionLength: 2
});
{% endhighlight %}
